# Hepatic alveolar echinococcosis mimicking cholangiocarcinoma: a case study

**DOI:** 10.1186/s12879-026-12892-9

**Published:** 2026-03-26

**Authors:** Yanfang Wang, Tiantian Dong, Dan Yang, Shaoce Xu, Fang Nie

**Affiliations:** 1 Department of Ultrasound, The Second Hospital & Clinical Medical School, Lanzhou University, Lanzhou, China; 2 Department of Pathology, The Second Hospital & Clinical Medical School, Lanzhou University, Lanzhou, China

**Keywords:** Alveolar echinococcosis, Hepatic neoplasms, Cholangiocarcinoma, Image, Diagnosis

## Abstract

**Background:**

Hepatic alveolar echinococcosis (HAE) is associated with significant disability and mortality. After infection, there is a prolonged asymptomatic latent period, and most patients present at a late stage when they seek medical treatment. The radiological features of HAE can resemble those of liver malignancies, posing diagnostic challenges.

**Case summary:**

A 52-year-old male was admitted to our hospital with discomfort and pain in the upper right abdomen persisting for more than six months without an apparent trigger. Abdominal ultrasound revealed a heterogeneous mass in the right posterior lobe of the liver, characterised by an irregular shape and indistinct boundaries. Enhanced ultrasound demostrated slight hyper-enhancement of the peripheral area during the arterial phase and hypo-enhancement in the delayed phase, while the central area showed no enhancement. CT and MRI scans revealed a large abnormal lesion in the right posterior lobe and porta hepatis of the liver, accompanied by marked dilation of the intrahepatic bile ducts and multiple enlarged lymph nodes at the porta hepatis. The lesion’s edges exhibited mild enhancement in the delayed phase, whereas the centre showed no significant enhancement. Serum CA199 levels were markedly elevated, exceeding the upper reference limit by more than sevenfold. The patient’s serum rEm18-ELISA test for alveolar cyst was negative. The pathological diagnosis was obtained via ultrasound-guided liver biopsy. The initial biopsy showed no clear evidence of tumour lesions. A repeat biopsy revealed hepatic alveolar echinococcosis, with Candida infection in the necrotic tissue. Four months later, the patient underwent surgical treatment at another hospital. Pathological examination confirmed hepatic alveolar echinococcosis, and the patient made a good recovery.

**Conclusion:**

Hepatic alveolar echinococcosis can closely mimic perihilar cholangiocarcinoma, exhibiting almost identical imaging features. Initial biopsies may yield false-negative results due to sampling necrotic tissue. Therefore, it is essential to consider the patient’s epidemiological background, maintain a high index of suspicion, and perform repeat biopsies to ensure an accurate diagnosis. Furthermore, it is crucial to inhance heathcare professionals’ proficiency in both clinical and technical aspects, promote effective interdepartmental communication, and reduce the incidence of misdiagnosis.

## Background

Alveolar echinococcosis is a zoonotic disease caused by the infection of humans and animals with the eggs of Echinococcus multilocularis. This parasite can infiltrate multiple organs within the human body, with the liver being the most commonly affected. Compared to cystic echinococcosis, which is caused by Echinococcus granulosus, alveolar echinococcosis is more pathogenic and is associated with higher rates of disability and mortality [1]. Research indicates that the 10-year mortality rate for untreated patients with hepatic alveolar echinococcosis (HAE) can be as high as 94% [2]. According to the World Health Organization, 91% of new HAE cases worldwide each year are reported in China [3]. Data from the China Centre for Disease Control and Prevention indicate that the mortality rate of the disease in China ranges from 50% to 75%, with the majority of cases occurring in pastoral regions of Xinjiang, Gansu, Tibet (Xizang), Ningxia, Sichuan, and other areas. Most patients have a history of residing in endemic regions or have had contact with animals such as cattle and sheep [4].

The progression of HAE is slow, proliferating through budding or infiltration, and continuously generating new vesicles that penetrate deeply into tissues. It not only has the capacity to invade surrounding normal liver tissue, adjacent bile ducts, and vascular systems, but can also spread extrahepatically to the retroperitoneum, lungs, brain, and other regions via lymphatic and blood channels, earning it the designation “worm cancer“ [5]. Despite HAE is classified as a benign tumour, it can sometimes be challenging to distinguish from malignant liver tumours through imaging. In this case report, we present an instance of HAE that mimicked cholangiocarcinoma and explore the potential causes of misdiagnosis.

## Case presentation

### Initial presentation and clinical findings

A 52-year-old male was admitted to our hospital on March 18, 2025, with a history of intermittent discomfort and pain in the upper right abdomen persisting for over six months without apparent triggers, which intensified postprandially. One week prior to admission, the patient developed jaundice affecting the skin and sclera, accompanied by fatigue. During the course of the illness, the patient experienced unexplained gastric pain seven months earlier, followed by the onset of fever four months ago. No other symptoms such as nausea, vomiting, acid reflux, belching, or a bitter taste were reported. Initially, these symptoms were not considered serious.

One month earlier, the patient sought medical attention at a local hospital, where an abdominal ultrasound revealed a hyperechoic lesion in the right posterior lobe of his liver, along with dilation of the intrahepatic bile ducts, rasing suspicion of liver lesions. An abdominal computed tomography (CT) scan showed a mixed-density lesion in the same region, significant dilation of the intrahepatic bile ducts, and poor visualization of the extrahepatic bile ducts. Additionally, multiple lymph nodes were detected in the porta hepatis, suggesting a possible liver malignancy with lymph node metastasis in this area.

The patient sought further diagnostic evaluation and treatment at our hospital and was admitted to the outpatient department with a diagnosis of “liver space-occupying lesions.” Since the onset of symptoms, the patient has remained conscious but has experienced a decline in mental state, appetite, and sleep quality, while maintaining normal bowel movements. The patient reported yellow urine and a recent weight loss of approximately 3 kg. He denied any history of hypertension, hepatitis, or residence in endemic areas but acknowledged a history of smoking and alcohol consumption.

### Physical examination and laboratory tests

The patient’s physical examination upon admission revealed the following observations: a body temperature of 36.5 ℃, a pulse rate of 56 beats per minute, a respiratory rate of 20 breaths per minute, and a blood pressure reading of 104/58 mmHg. Laboratory tests produced the following results: an alanine aminotransferase level of 157 U/L, positive urobilin and urobilinogen tests, an eosinophil ratio of 8.5%, a lymphocyte count of 0.80 × 10^9^/L, an alpha-fetoprotein level of 2.92 ng/mL, a carcinoembryonic antigen level of 2.52 ng/mL, a carbohydrate antigen CA125 level of 12.1 U/mL, and a carbohydrate antigen CA199 level of 209 U/mL. The patient exhibited no signs of infectious disease, and coagulation function was normal.

### Imaging work-up (US/CEUS, CT, MRI)

**Fig. 1:**
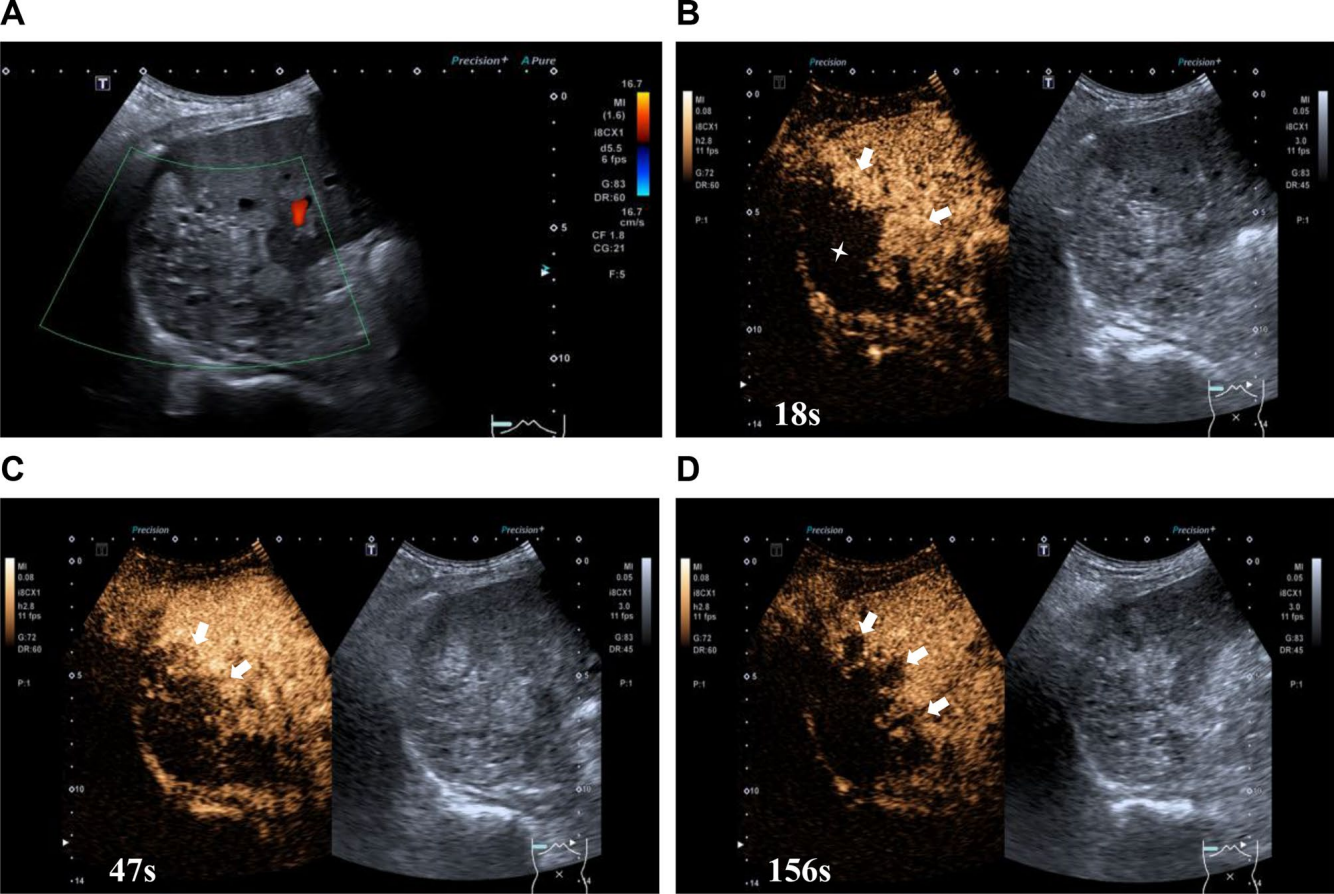
Ultrasound findings. (**A**) Conventional ultrasound revealed a heterogeneous mass in the right lobe of the liver. (**B**) Contrast-enhanced ultrasound showed the periphery of the lesion was slightly hyper-enhanced compared to the surrounding normal liver tissue at 18 s after contrast agent injection (arrow), with a blurred boundary and no enhancement in the interior of the lesion (asterisk). (**C**) Contrast-enhance ultrasound at 47s after contrast agent injection revealed that the contrast agent began to wash out at the periphery of the lesion (arrow). (**D**) Contrast-enhance ultrasound at 156s after contrast agent injection showed the periphery of the lesion was hypo-enhancement (arrow) and no enhancement in the inner part

A conventional ultrasound examination identified a mixed mass measuring 10.3 × 7.6 × 7.0 cm in segments 6 and 7 of the liver, characterised by an irregular shape and indistinct margins (Fig. 1A). The periphery of the lesion exhibited slight hyper-enhancement compared to the surrounding normal liver tissue during the arterial phase (AP) following administration of the SonoVue contrast agent (Fig. 1B). It began to exhibit wash out during the early portal vein phase (PVP) (Fig. 1C), and continued to wash out during the late PVP and delayed phases (DP), displaying hypo-enhancement (Fig. 1D). Meanwhile, the internal region remained unenhanced throughout these three phases, and the lesion’s boundaries remained indistinct (Fig. 1B and D). Ultrasound findings suggested an enhancement pattern indicative of a malignant lesion, raising suspicion cholangiocarcinoma, and a biopsy was recommended.

**Fig. 2:**
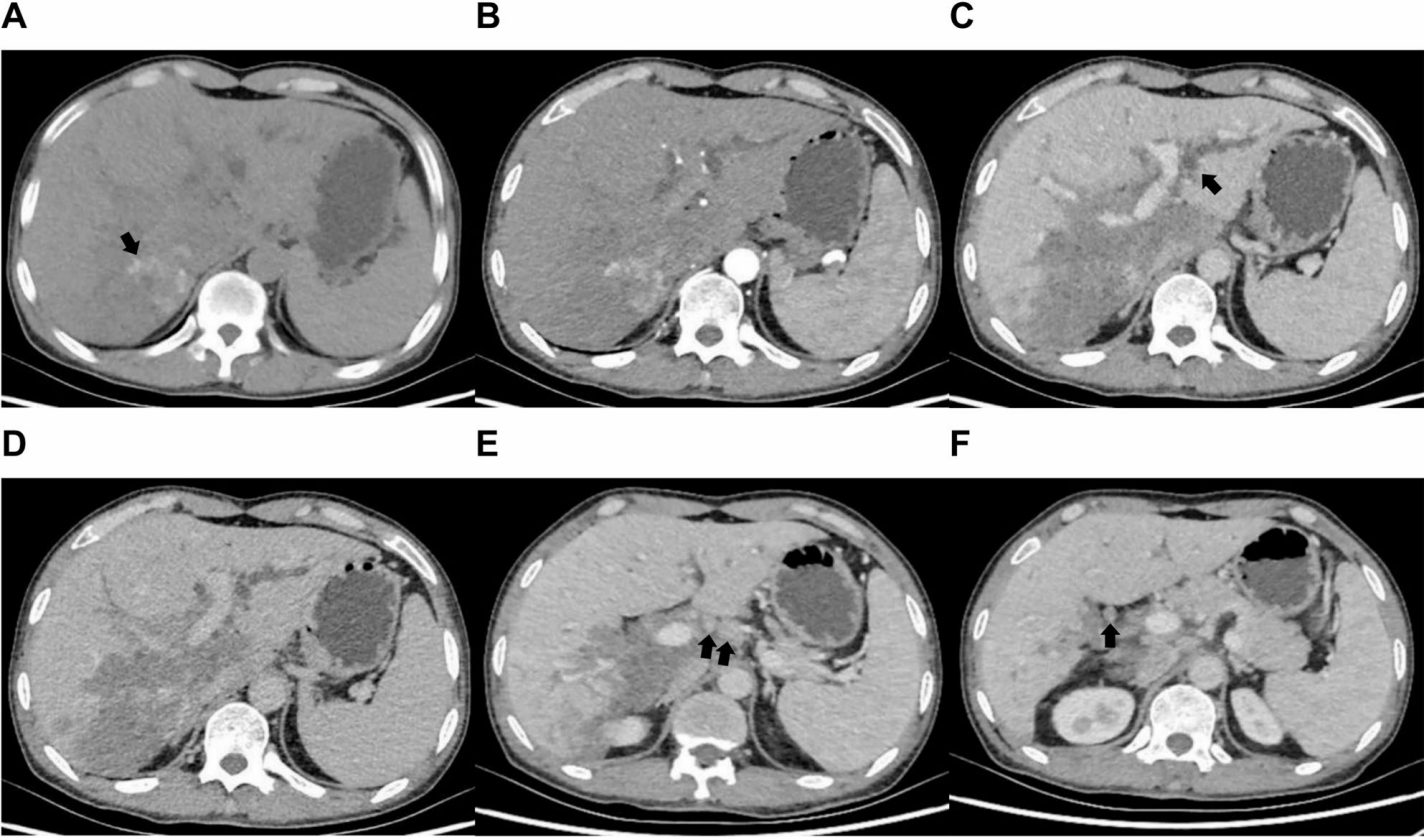
CT findings. (**A**) The plain CT scan revealed a substantial mixed low-density area in the right lobe of the liver, with hyperdense foci (arrow) within the lesion and encasement of both the right and left portal. (**B**,** C**,**D**) The arterial phase (**B**), portal veous phase (**C**), and delayed phase (**D**) of the lesion on contrast-enhanced CT demonstrated no evident enhancement within the lesion; however, there was notable dilation of the intrahepatic bile ducts (arrow). (**E**,** F**) The contrast-enhanced CT images at the level of the porta hepatis show multiple enlarged perihilar lymph nodes (arrows)

CT image revealed a large, mixed low-density area in the right lobe of the liver, measuring approximately 6.5 × 11.2 × 8.0 cm. Hyperdense foci were present within the lesion, which encased both the right and left portal veins (Fig. 2A). The CT attenuation values within the lesion were 44, 44, 46, and 46 Hounsfield Units (HU) on the non-contrast and each enhanced phase, respectively (Fig. 2A and D). The intrahepatic bile ducts were markedly dilated, and stenosis was noted in the right branch of the portal vein. Additionally, the wall of the common bile duct was thickened, with luminal narrowing. A small nodular enhancing shadow was identified in the upper segment, with CT values of 34, 52, 69, and 84 HU. Furthermore, multiple enlarged lymph nodes were observed in the porta hepatis (Fig. 2E and F), demonstrating moderate enhancement. The CT findings suggested atypical liver enhancement, with a suspected diagnosis of cholangiocarcinoma and possible multiple metastatic lymph nodes in the porta hepatis.

**Fig. 3:**
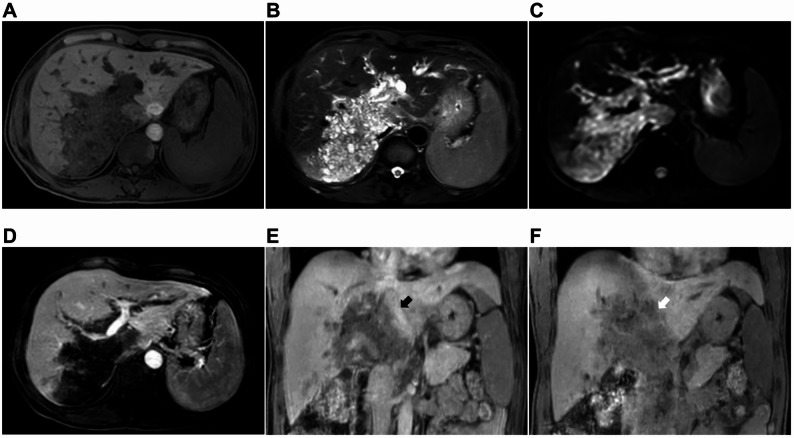
MRI findings. (**A**,** B**,**C**) The MRI plain scan revelaed a substantial lesion in the right posterior lobe of the liver and porta hepatis, appearing hypointense on T1WI and hyperintense on T2WI and DWI. The lesion showed periportal infiltration, encasing the right and left portal veins, and invading of the caudate lobe. (**D**) The enhanced MRI demonstrated no enhancement of the lesion during the arterial phase. (**E**,** F**) Mild local enhancement (black arrow) was observed at 3 min after contrast agent injection; however, the lesion exhibited hypointensity (write arrow) at 15 min post-injection

The magnetic resonance imaging (MRI) examination revealed a large hypointense on T1-weighted imaging (T1WI) and hyperintense area on T2-weighted imaging (T2WI) within the right posterior lobe of the liver and the porta hepatis, characterised by an irregular shape and indistinct boundaries (Fig. 3A and B). Diffusion-weighted imaging (DWI) also demonstrated a mildly elevated signal (Fig. 3C). The mass demonstrated extensive periportal infiltration around the right and left portal veins and extended into the caudate lobe (Fig. 3A and C). During enhanced scanning, no significant enhancement was observed in the AP (Fig. 3D) and PVP; however, mild local enhancement was noted at 3 min, with hypointensity evident at 15 min (Fig. 3E and F). Dilation of the bile duct in the left lobe was observed, although there was no significant dilation of the extrahepatic bile duct. Additionally, multiple enlarged lymph nodes were identified in the porta hepatis. Based on the MRI findings, cholangiocarcinoma (BC type IV) with lymph node metastasis in the porta hepatis was suspected. Following this examination, and in conjunction with the patient’s medical history and physical examination, a preliminary clinical diagnosis of cholangiocarcinoma was made, and a liver tissue biopsy was recommended.

### First biopsy and pathology findings

**Fig. 4:**
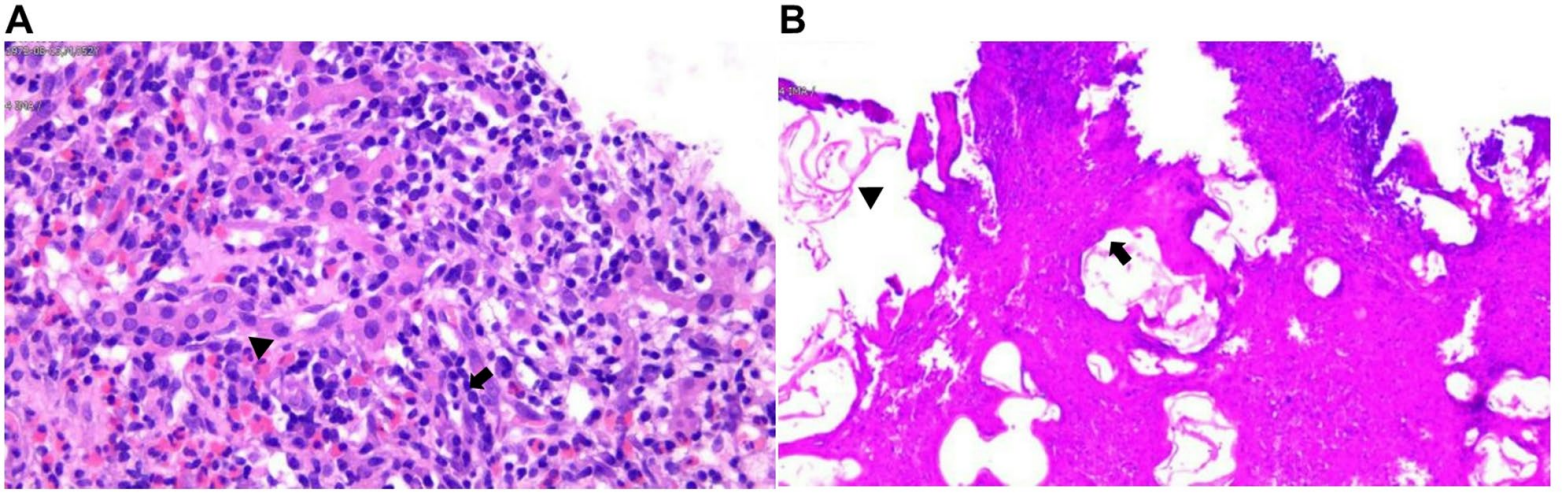
Histopathological findings of the two liver biopsies. (**A**) First biopsy: H&E-stained sections showing a notable presence of lymphocytic (arrows) and eosinophils (arrowheads) infiltration within the hepatic tissue, with no tumour cells or parasitic structures observed (×400). (**B**) Second biopsy: H&E sections demonstrating fibrous tissue with hyaline degeneration, inflammatory cells, and granulomatous nodules, accompanied by eosinophilic laminated parasitic membranes (arrows) and fungal hyphae (arrowheads), confirming hepatic alveolar echinococcosis with Candida co-infection (×100)

On 26 March 2025, a liver tissue biopsy was performed in the interventional ultrasound department, targeting the peripheral area with the enhanced with SonoVue agent for sampling. The tissue specimens were white, measuring approximately 1.8 cm and 1.6 cm in length, respectively. Pathological examination revealed two puncture tissue samples: one contained necrotic material, while the other showed lymphocytic infiltration and extensive eosinophilic infiltration in the hepatic portal tracts, accompanied by intrahepatic cholestasis and inflammatory changes. No definitive neoplastic lesions were identified. A repeat biopsy is recommended when clinically indicated (Fig. 4A). At this point, the clinicians considered the possibility of HAE. Upon further detailed inquiry into the patient’s history, it was revealed that patient had resided in Gannan (a pastoral area) for one week many years previously due to work commitments, a fact he had initially forgotten. A serum test for alveolar cysts using rEm18-ELISA was performed, yielding a negative result.

### Repeat biopsy, final diagnosis, and subsequent management

Given the limited sample collection and the absence of identifiable tumour cells or parasitic structures, a repeat biopsy was performed on 31 March 2025, to further clarify the diagnosis. Two punctures were made at the margin of the tumour-enhanced area, each yielding specimens measuring 1.8 cm in length. Subsequent pathological examination revealed fibrous tissue with hyaline degeneration, inflammatory cells, and granulomatous nodules within the hepatic portal tracts, accompanied by eosinophilic laminated parasitic membranes. Additionally, branching, septate fungal hyphae were identified within necrotic areas, confirming a concurrent Candida species infection (Fig. 4B). These findings led to a definitive diagnosis of HAE with Candida infection.

The patient and their family were informed of the diagnosis and the proposed treatment plan. The family indicated their understanding of the situation, declined further treatment, and requested discharge. In a follow-up telephone call six months later, we learned that the patient had undergone surgical treatment at another hospital four months after discharge. The pathological results confirmed the diagnosis of HAE, and the patient reported a good recovery.

## Discussion

HAE, although uncommon, is a potentially fatal parasitic infection, if left untreated. Consequently, accurate diagnosis of HAE is of considerable importance. This case study, involving a 52-year-old male patient with a hepatic lesion identified on imaging, illustrates the atypical presentation and diagnostic challenges associated with HAE. The exceptional value of this case lies in two aspects: (1) the striking radiological resenblance to perihilar cholangiocarcinoma across multiple imaging modalities (CT, MRI, CEUS); and (2) the diagnostic challenge posed by an initially negative biopsy and serological tests, with the diagnosis of HAE only confirmed upon repeat sampling.

In its early stages, HAE often lacks distinct clinical symptoms. As the disease progresses, the lesion may enlarge, invade surrounding vascular structures, and compress adjacent tissues, leading to symptoms such as upper abdominal distension and pain, obstructive jaundice, portal hypertension, liver failure, cachexia, and involvement of other organs, including the lungs or brain [6]. The presence of pronounced clinical symptoms typically indicates progression to PNM

Stage Ⅱ–Ⅳ of HAE [7]. According to the guidelines of Brunetti E et al. [6], the patient in this case exhibited no significant symptoms during the early stages. However, as the mass enlarged in later stages, the patient experienced intermittent pain and discomfort in the upper right abdomen. One week prior to presentation, the patient developed jaundice, weakness, and experienced a weight loss of 3 kg, which consistent with the clinical manifestations of advanced HAE. Furthermore, the patient’s history of residing in Gannan is noteworthy, as it aligns with the epidemiological characteristics typical of HAE and aids diagnosis.

Currently, the diagnosis of HAE primarily relies on imaging techniques, with serological testing serving as an important supplementary tool [8]. The imaging modalities most commonly employed include abdominal ultrasound, CT, and MRI. The clinical manifestations of HAE often lack distinctive features, which can lead to misdiagnosis as cholangiocarcinoma, hepatocellular carcinoma, or hepatic haemangioma, among other conditions. Ultrasound has become the preferred imaging modality for the screening and diagnosis of hepatic echinococcosis due to its advantages of being non-radiative, cost-effective, and rapid. CT imaging accurately localises hepatic echinococcal cysts and delineates the anatomical and morphological characteristics of echinococcal lesions. MRI is particularly valuable in clarifying the relationship between bile ducts and lesions and can clearly depict the small cystic structures associated with the alveolar larval stages, thereby facilitating the definitive diagnosis of HAE [9].

Typical imaging features of HAE include a heterogeneous, infiltrative mass with indistinct margins on conventional ultrasound, often exhibiting calcifications (the “hailstorm” pattern) or a thick (> 10 mm) hyperechoic rim surrounding a necrotic center (the “worm-eaten” appearance) [10, 11]. On CEUS, HAE commonly demonstrates peripheral rim-like or irregular enhancement from the AP through the PVP and DP, with a non-enhancing central core (the “black hole sign”) [11, 12]. CT usually reveals an irregular, low- or mixed-density mass with internal calcifications, central necrosis (the “map-like” appearance), and mild delayed marginal enhancement. Mild peripheral enhancement may be observed following contrast administration, whereas the central region generally remains unenhanced. The presence of small vesicles is considered a relatively specific imaging feature of HAE [13, 14]. MRI typically shows hypointensity on T1WI, hyperintensity on T2WI. and absent or delayed ring-like enhancement [15].

In the present case, conventional ultrasound revealed a calcified hepatic mass with irregular morphology, scattered echogenic foci, and small internal hypoechoic areas. CEUS demonstrated peripheral “worm-eaten” enhancement, which began to wash out during the early portal venous phase and became markedly hypo-enhanced in the delayed phase, while the central region remained unenhanced. CT showed a large right-lobe mass with mixed low density (non-contrast HU: 44; arterial/portal/delayed phases: 44, 46, 46 HU), with no significant enhancement across all phases, and enlarged lymph nodes at the porta hepatis. MRI revelaed a well-defined lesion that was hypointense on T1WI, and hyperintense on T2WI and DWI. There was no appreciable enhancement in the arterial or portal phases, with only focal mild enhancement at 3 min and wash out at 15 min. Additionally, dilation of the left hepatic duct was observed.

This constellation of findings diverged from classic HAE in several key aspects: (1) the early washout and marked hypo-enhancement on CEUS during the delayed phase—unlike the persistent peripheral enhancement typical of HAE; (2) stable CT attenuation without progressive enhancement, contrasting with the expected delayed marginal enhancement; (3) porta hepatis lymphadenopathy and contralateral bile duct dilation, features highly suggestive of cholangiocarcinoma but rare in uncomplicated HAE. Although the central non-enhancement and calcifications were compatible with HAE, the overall imaging phenotype closely mimicked perihilar cholangiocarcinoma. We attribute the transient peripheral enhancement on CEUS to inflammatory hyperaemia and neovascularisation within the reactive fibrotic rim, rather than neoplastic angiogenesis. This case underscores that atypical imaging presentations of HAE can masquerade as malignancy, necessitating the integration of serology, targeted biopsy, and clinical context for accurate diagnosis.

Serological testing is crucial for the diagnosis, epidemiological investigation, and monitoring of HAE [9]. The Em18 antigen has demonstrated high sensitivity and specificity for alveolar echinococcosis and has been employed in ELISA and immunoblotting techniques [16, 17]. Recently, an enzyme immunochromatography test using the recombinant Em18 (rEm18-ELISA) antigen has been developed, offering a sensitivity of 98.0% and a specificity of 99.3% for HAE [18]. Furthermore, the rEm18-ELISA currently represents the most effective serological assay for monitoring the therapeutic progres of alveolar echinococcosis [19]. Nonetheless, it is important to note that positive serology alone is insufficient to definitively diagnose the disease, as serological results may be negative even in histologically confirmed AE cases [19, 20]. In the present case, the patient’s serum test for rEm18-ELISA targeting the alveolar cyst was negative. Analysis of the possible causes suggests: (i) the predominantly necrotic or fibrotic nature of the lesion, which can limit antigen release and subsequent antibody production; (ii) an early or loccalised disease stage, where the host immune response has not yet generated detectable levels of Em18-specific IgG; (iii) individual variability in the humoral immune response, as observed in up to 10–15% of confirmed HAE cases [21]. In such scenarios, clinicians should prioritise tissue diagnosis via biopsy—ideally targeting the viable peripheral rim—and integrate multimodal imaging findings rather than relying solely on serology. Serological testing remains a valuable adjunct, but its limitations must be recognised within the diagnostic algorithm. In recent years, the rapid advancement of molecular diagnostic technologies has brought metagenomic next-generation sequencing (mNGS) to the forefront as a promising tool for identifying the etiology of infectious diseases [22]. In a study by Özdemir et al. [23], serum-derived exosomal circular RNAs (circRNAs) were analysed using high-throughput sequencing techniques. The findings revealed a 100% detection rate of circRNAs from Echinococcus granulosus in AE patients, while non-AE patients and healthy volunteers tested negative. This suggests that circRNAs from Echinococcus granulosus could serve as a potential biomarker for diagnosing HAE. Due to the unavailability of NGS technology at our facility, NGS testing was not conducted in this instance.

The definitive diagnosis of HAE depends on the histopathological examination of biopsied or surgically excised tissue. Under high-magnification microscopy, the primary diagnostic criterion is the identification of microcystic structures and lamellar bodies against a necrotic background [9]. Previous studies have indicated that lamellar bodies can be observed within the solid necrosis of alveolar echinococcosis lesions and in the adjacent liver tissue [24]. In the present case, the initial biopsy was obtained from the sub-central portion of the lesion, yieldeding scant tissue fragments cmposed predominantly of necrotic debris and inflammatory cells, with no identifiable tumour cells or parasitic structures. Routine H&E staining revelaed only chronic inflammation, and no special stains (e.g., PAS, GMS) were initially requested, as the clinical suspicion at that time strongly favoured toward cholangiocarcinoma. However, the second biopsy was deliberately directed towards the enhancing peripheral rim of the mass. This yielded adequate viable tissue, revealing characteristic laminated parasitic membranes consistent with Echinococcus multilocularis. Additional PAS stainning confirmed fungal elements (Candida spp.). The pathologist’s initial differential diagnosis included cholangiocarcinoma and abscess, but HAE was not strongly considered until clinicians and imaging correlation prompted a re-evaluation and targeted pathological slide review.

In analysing this case, the primary factors contributing to misdiagnosis include the following: firstly, the imaging manifestations of HAE close resemble those of cholangiocarcinoma. Conventional ultrasound examination identified a substantial hyperechoic region within the right hepatic lobe, characterised by multiple scattered calcifications and small cystic anechoic areas that were poorly delineated from the adjacent liver parenchyma. Additionally, intrahepatic bile duct dilation was noted. Contrast-enhanced ultrasound (CEUS) demonstrated a ‘fast-in-fast-out’ enhancement pattern at the periphery of the lesion, with no enhancement observed within the lesion itself, potentially indicative of infiltrative growth. Enhanced CT revealed mild enhancement of the lesion, pronounced dilation of the intrahepatic bile ducts, and multiple lymph nodes in the porta hepatis exhibiting moderate enhancement on contrast-enhanced scanning, possibly attributable to a local inflammatory response surrounding the liver. MRI showed that the lesion exhibited enhancement during the early DP, which diminished to low signal intensity in the late DP, possibly due to partial contrast agent leakage following liquefactive necrosis of the lesion. These imaging characteristics closely resemble those associated with cholangiocarcinoma, which are infrequently observed in the typical imaging features of HAE. Secondly, the patient’s epidemiological history was not given sufficient consideration. Although he had resided in a non-endemic area for an extended period, he had previously lived in Gannan, a region recognised as high-risk for echinococcosis. The attending physician did not conduct a thorough investigation into the patient’s epidemiological background. Thirdly, there was a limited comprehension of HAE. The imaging features of HAE are atypical, and serological tests may produce false-negative results. Hepatobiliary surgeons based their diagnoses on imaging findings and negative serological test results for echinococcosis, wihtout undertaking a comprehensive analysis of HAE or considering differential diagnoses from similar diseases.

In conclusion, HAE must be considered in the differential diagnosis of perihilar masses in endemic regions, even when imaging strongly suggests cholangiocarcinoma. A negative initial biopsy and serological test in a suspicious mass should not exclude HAE; a repeat biopsy targeting viable peripheral tissue is warranted. The patient’s epidemiological background is also very important. Furthermore, it is essential to enhance the expertise of healthcare professionals in both clinical and medical-technical fields, promote effective interdepartmental communication, and reduce the incidence of misdiagnosis.

## Data Availability

The data used during the current study are available from the corresponding author.

## Abbreviations

HAE: Hepatic alveolar echinococcosis
CEUS: Liver contrast-enhanced ultrasound
CT: Computed tomography
MRI: Magnetic resonance imaging
AP: Arterial phase
PVP: Late portal venous phase
LP: Late phase
T1WI: T1-weighted image
T2WI: T2-weighted image
DWI: Diffusion-weighted imaging
mNGS: Metagenomic next-generation sequencing
circRNAs: Circular RNAs
